# Stroke-Like Migraine Attacks After Radiation Therapy (SMART) Syndrome: A Diagnostic Challenge a Decade After Cranial Irradiation

**DOI:** 10.7759/cureus.98494

**Published:** 2025-12-04

**Authors:** Akshayaa K Aggarawal, Nang Soe Yamin Mon, Mansoor Gazi

**Affiliations:** 1 Internal Medicine, Walsall Manor Hospital, Birmingham, GBR; 2 Acute Internal Medicine, Walsall Manor Hospital, Walsall, GBR

**Keywords:** brain scans, epileptic seizures, episodic migraine, neuro oncology, stroke

## Abstract

Stroke-like migraine attacks after radiation therapy (SMART) syndrome is a rare, late complication of cranial radiotherapy characterized by recurrent neurological symptoms, which may include headaches, hemiparesis, aphasia, seizures and confusion. The condition typically manifests years after radiation exposure, with magnetic resonance imaging (MRI) showing transient, reversible cortical abnormalities in previously irradiated regions. Although often self-limiting, neurological recovery may be incomplete in some cases.

We present the case of a 64-year-old elderly male with a prior history of cranial irradiation received in 2015 who was admitted with abnormal body movements. Initial blood investigations and a non-contrast CT head were unremarkable. The MRI brain scan demonstrated deep white matter ischemic changes with no new focal lesions compared to a prior scan. Cerebrospinal fluid analysis was normal. Electroencephalography revealed focal onset seizures with secondary generalization. Following neurology review, antiepileptic therapy was optimized. Based on the clinical history, radiological findings, and seizure disorder, a diagnosis of SMART syndrome was made. The patient gradually improved his level of consciousness with a decrease in seizure activity with good neurological recovery.

## Introduction

Stroke-like migraine attacks after radiation therapy (SMART) syndrome is a rare, delayed complication of cranial irradiation, characterized by transient and occasionally recurrent neurological deficits. The condition was first described in children by Shuper and colleagues in 1995, who reported complicated migraine-like episodes in patients experiencing severe, intermittent unilateral headaches accompanied by nausea several years after receiving cranial irradiation and chemotherapy. Importantly, these episodes did not necessarily indicate stroke, vasculitis, or tumor recurrence, although their clinical presentation commonly mimics these conditions. While cerebral angiography may aid in differentiating SMART syndrome from other pathologies, its use has been associated with transient exacerbation of symptoms [1].

Fewer than 150 cases have been reported globally, underscoring both the rarity of SMART syndrome and the diagnostic challenges it poses [2,3]. Patients typically present years to decades after cranial irradiation with a constellation of symptoms, including headaches, seizures, visual disturbances, hemiparesis, aphasia, and migraine-like episodes. The latency between radiation exposure and symptom onset is highly variable, ranging from as early as one year to as long as 37 years [4,5]. Although the diagnosis traditionally relies on the reversibility of clinical and radiological findings, Black et al. reported in 2013 that a substantial proportion of patients may experience incomplete recovery, challenging earlier assumptions about its benign course [6]. Prior to confirming SMART syndrome, alternative etiologies such as local tumor recurrence, leptomeningeal involvement, and ischemic pathology must be rigorously excluded [7].

The exact pathophysiology of SMART syndrome remains incompletely understood. Several mechanisms have been proposed, including radiation-induced vascular dysfunction, delayed endothelial injury, mitochondrial impairment, and abnormal cortical excitability, which collectively may lead to impaired cerebral autoregulation and cortical spreading depression [3,8]. Following cranial irradiation, late delayed effects within the central nervous system are believed to arise from both parenchymal and vascular injury involving oligodendrocytes, neural progenitor cells, and endothelial structures. Progressive late-onset damage after high-dose radiation has been attributed to the generation of long-lived free radicals, reactive oxygen species, and pro-inflammatory cytokines, contributing to chronic neuroinflammation and tissue dysfunction [9].

Characteristic neuroimaging findings are central to establishing the diagnosis of SMART syndrome. Magnetic resonance imaging (MRI) typically demonstrates reversible, unilateral gyriform cortical gadolinium enhancement with associated T2/FLAIR hyperintensity, often with relative sparing of the subcortical white matter [2,10]. Karmen Wai et al. further reported that SMART syndrome may show pronounced hypoperfusion on hyperacute CT perfusion (CTP) imaging without subsequent infarction [11]. Although these imaging features are not pathognomonic, they are valuable in differentiating SMART syndrome from other important mimics such as tumor recurrence, infectious encephalitis, posterior reversible encephalopathy syndrome (PRES), and acute ischemic stroke [3,12].

Epidemiological reviews suggest a mean age of onset in the mid-40s, though cases have been reported in both pediatric and elderly populations, reflecting the wide clinical spectrum [13,14]. The condition is typically associated with prior high-dose cranial radiotherapy (>50 Gy), but cases with lower doses have been described [3,4]. Although most patients experience spontaneous recovery within weeks to months, up to one-third may suffer persistent neurological sequelae or recurrent episodes in almost half of the patients [5,14].

Currently, there is no standardized treatment protocol. Corticosteroids, antiepileptic drugs, and supportive care remain the mainstay of management, with emerging but still preliminary evidence suggesting potential benefit from L-arginine therapy due to its role in improving endothelial and mitochondrial function [15]. Accurate recognition of this syndrome is crucial as misdiagnosis can lead to unnecessary invasive procedures or delayed treatment, adversely affecting patient outcomes [3,10].

In this report, we describe a rare case of SMART syndrome, adding to the growing body of literature on this unusual but clinically significant post-radiation complication.

## Case presentation

A 63-year-old male presented with the acute onset of left-sided weakness affecting both the upper and lower limbs, accompanied by a headache that had developed earlier the same day. He reported a one-year history of recurrent headaches that had progressively increased in frequency, typically lasting 5-10 minutes, of moderate-to-severe intensity, and occasionally relieved with medication. On arrival, he was drowsy with a Glasgow Coma Scale (GCS) score of E2V3M4, indicating eye opening to pain, inappropriate verbal responses, and withdrawal to painful stimuli. His vital signs were within normal limits: blood pressure 124/76 mmHg, heart rate 80 beats per minute, respiratory rate 16 breaths per minute, normal oxygen saturation on room air, and no pyrexia.

Neurological examination demonstrated a left upper motor neuron (UMN) facial palsy, with motor weakness graded 3/5 in the left upper and lower limbs, and an upgoing plantar response on the left, consistent with a UMN lesion. Sensory examination was unremarkable. Muscle tone and deep tendon reflexes on the left side were normal. The right upper and lower limbs demonstrated normal power, tone, and reflexes, with a downgoing plantar response. Cerebellar assessment could not be reliably performed.

The patient’s past medical history was notable for a right temporal lobe anaplastic oligodendroglioma, WHO grade III, with 1p/19q codeletion, diagnosed in 2015. He had undergone cranial radiotherapy to a total dose of 54 Gy in 30 fractions over two months, adjusted for tumor volume, followed by five cycles of adjuvant PCV chemotherapy (procarbazine, lomustine, and vincristine). His additional comorbidities included type 2 diabetes mellitus, dyslipidemia, and focal epilepsy.

During the current hospitalization, he experienced recurrent generalized tonic-clonic seizures, each lasting approximately five minutes. Between episodes, he failed to regain full consciousness, raising concern for convulsive status epilepticus. There was no associated tongue biting or urinary or fecal incontinence. These seizures occurred several times daily and were witnessed by clinical staff. Each episode was followed by a period of post-ictal confusion. Lorazepam was administered during these events, resulting in clinical improvement. Over the following days, the patient regained full consciousness and returned to his baseline neurological status. He improved after neurology reviewed and optimised the treatment. 

The case was reviewed regularly with the neurology team to optimize antiepileptic management. His prior therapy with verapamil was discontinued as it was ineffective in controlling seizure activity. A trial of valproate was initiated and continued for one week but subsequently withdrawn due to insufficient seizure control. Lacosamide was then introduced, resulting in significant clinical improvement and effective seizure suppression. The initial neurological plan included comprehensive investigations: a non-contrast CT brain, MRI brain, EEG, and cerebrospinal fluid (CSF) analysis.

**Figure 1:**
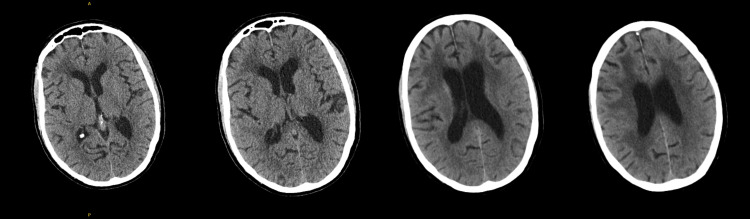
CT head showing no acute pathology Non-contrast axial CT images of the brain show no evidence of acute intracranial hemorrhage or large territorial infarction.

Neuroimaging was performed at several time points during admission. The non-contrast CT brain on arrival demonstrated chronic small-vessel ischemic changes and age-appropriate cerebral atrophy, with no evidence of acute hemorrhage, infarction, or mass lesion. A repeat non-contrast CT performed 10 days later showed unchanged findings, revealing mild arteriosclerotic leukoencephalopathy without any acute intracranial pathology (Figure 1).

**Figure 2:**
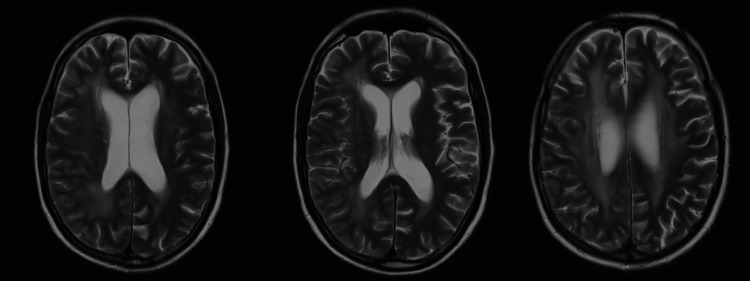
MRI head showing chronic small-vessel ischemia The MRI brain shows global cerebral atrophy, extensive periventricular hyperintensities consistent with chronic small-vessel ischemia, and no evidence of acute infarction or mass lesion

The MRI of the brain on admission revealed global cerebral atrophy, extensive periventricular hyperintensities consistent with chronic small-vessel ischemia, and no evidence of acute infarction or mass lesion. A follow-up MRI after 10 days demonstrated unchanged findings, again without evidence of recurrent tumor or acute ischemia (Figure 2).

**Figure 3:**
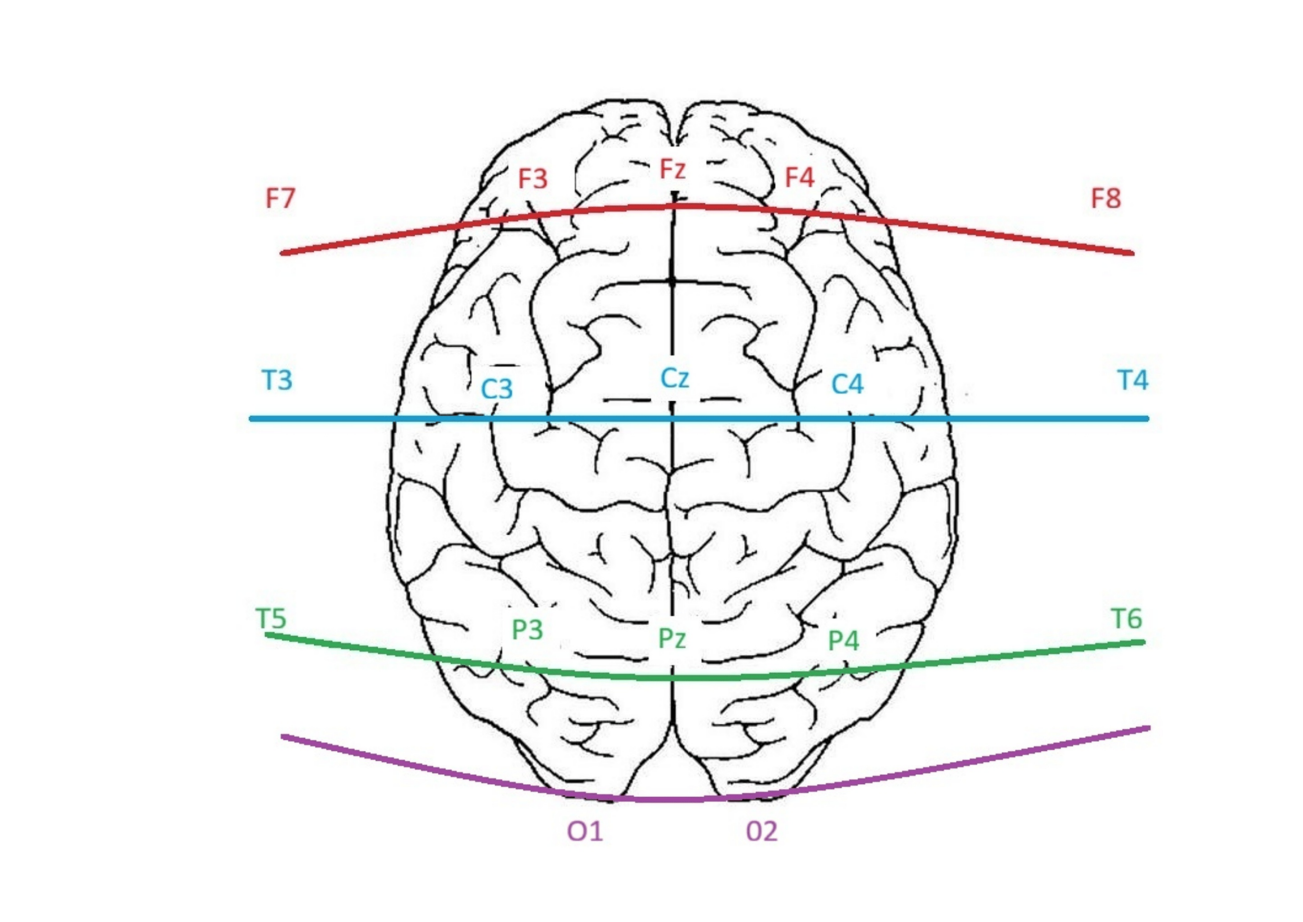
The bipolar EEG montage. The figure demonstrates a bipolar EEG montage used in the study. Lettered labels denote underlying brain regions (F-frontal, C-central, P-parietal, T-temporal, O-occipital), while odd/even numbers correspond to left and right hemispheres, respectively; Z indicates midline placement. Electrodes are arranged in longitudinal chains across frontal, central, and parietal regions. Each bipolar channel reflects the voltage difference between two neighbouring electrodes, enabling localization of cortical activity. Created by authors.

**Figure 4:**
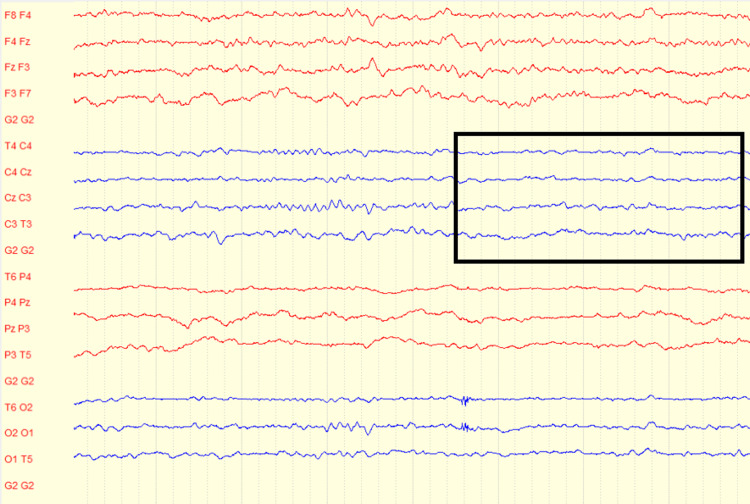
The EEG recording demonstrating focal slowing in a bipolar montage. The EEG segment shows slowing over the right hemisphere, most notable in the central and temporal chains (T4-C4, C4-Cz, Cz-C3, C3-T3) (outlined in black). This slowing reflects focal cortical dysfunction correlating with the patient’s known prior right-hemispheric pathology. No clear epileptiform discharges (sharp waves or spikes) are seen within this time frame.

**Figure 5:**
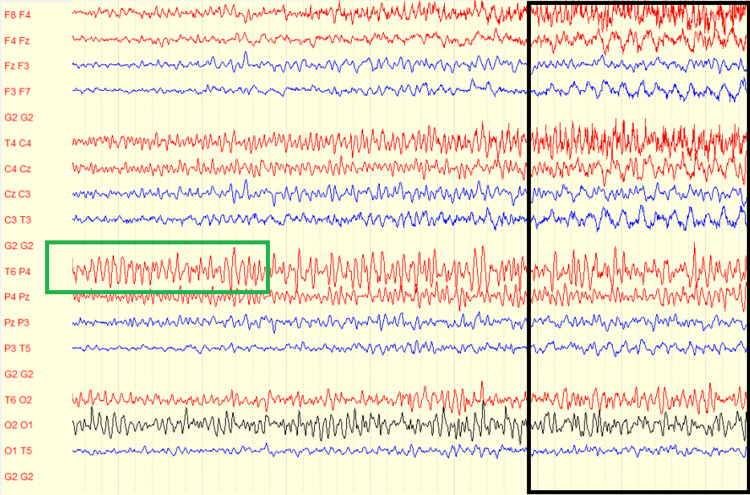
EEG demonstrating focal-onset seizure with secondary generalization in a bipolar montage. The EEG shows an increase in rhythmicity and amplitude in the right posterior temporal-parietal region. T6-P4 showing the origin of seizure activity (outlined in green). This seizure activity then evolves in both hemispheres (outlined in black). This evolution indicates secondary generalization, consistent with clinical manifestations of generalized tonic-clonic activity.

Electroencephalography (EEG) performed on admission demonstrated slowing over the right hemisphere, consistent with his prior history of right temporal oligodendroglioma. During a recorded clinical seizure episode characterized by mouth and head deviation, the EEG revealed a build-up of faster activity over the right hemisphere that subsequently spread to the left hemisphere. These findings supported a diagnosis of focal onset seizures with secondary generalization (Figures 3-5).

Cerebrospinal fluid (CSF) analysis revealed clear and colorless fluid with normal glucose and protein concentrations, four white blood cells, and no red blood cells. Oligoclonal bands were absent, and CSF cultures and PCR assays for common viral and bacterial pathogens were negative (Table 1).

**Table 1:** Cerebro spinal fluid (CSF) analysis.

Test	Value	Normal value
CSF glucose	4.4 mmol/L	2.7-4.4 mmol/L
Oligoclonal banding	Not seen	Not present
Appearance	Clear, colourless	Clear, colourless
White blood cells	4 cells/µL	<5 cells/µL
Red blood cells	0 cells/µL	0 cells/µL
Organisms	No organisms seen	None seen
Culture	No growth	No growth
Aerobic bottle	No growth after 5 days incubation	No growth
Anaerobic bottle	No growth after 5 days incubation	No growth

Comprehensive paraneoplastic antibody testing of the CSF, including collapsin response mediator protein 5 (CV2/CRMP5), para-neoplastic Ma family member 2 (Ma2/Ta), amphiphysin, Ri, Yo, Hu, SRY-box transcription factor 1 (SOX1), titin, zinc finger protein of the cerebellum 4, (ZIC4), glutamic acid decarboxylase 65 (GAD65), and Tr antigen-delta/notch-like epidermal growth factor-related receptor (Tr-DNER), yielded negative results (Table 2).

**Table 2:** Para neoplastic screening of CSF. CV2/CRMP5: collapsin response mediator protein 5, Ma2: para-neoplastic Ma family member 2, SOX1: SRY-box transcription factor 1, ZIC4: zinc finger protein of the cerebellum 4, GAD 65: glutamic acid decarboxylase 65, Tr (DNER): Tr antigen-delta/notch-like epidermal growth factor-related receptor.

Test	Result
Anti-CV2/CRMP5	Negative
Anti-Ma2	Negative
Anti-amphiphysin	Negative
Anti-Ri	Negative
Anti-Yo	Negative
Anti-Hu	Negative
Anti-recoverin	Negative
Anti-SOX1	Negative
Anti-Titin	Negative
Anti-ZIC4	Negative
Anti-GAD 65	Negative
Anti-Tr (DNER)	Negative

Likewise, serum autoimmune encephalitis panel, encompassing N-methyl-D-aspartate (NMDA), anti-α-amino-3-hydroxy-5-methyl-4-isoxazolepropionic acid receptor (AMPA), leucine-rich glioma-inactivated 1 antibodies (LGI1), gamma-aminobutyric acid type B receptor subunit 1/2 (GABA-B 1/2), and contactin-associated protein-like 2 (CASPR2) antibodies, were also negative (Table 3).

**Table 3:** Auto immune screening of serum. AMPA: anti-α-amino-3-hydroxy-5-methyl-4-isoxazolepropionic acid receptor, LG1L antibodies: leucine-rich glioma-inactivated 1 antibodies, GABA-B 1/2: gamma-aminobutyric acid type B receptor subunit 1/2, CASPR2: contactin-associated protein-like 2.

Test	Value
AMPA1 receptor antibody	Negative
AMPA2 receptor antibody	Negative
LG1L antibodies	Negative
N-methy-D-asparate (NMDA) receptor antibody	Negative
GABAB1/2 receptor antibody	Negative
CASPR2 antibodies	Negative

The patient’s clinical progression, radiological findings, and prior oncological history collectively guided the diagnostic evaluation. Serial neuroimaging showed no evidence of acute ischemia or tumor recurrence, while cerebrospinal fluid and serological studies remained entirely unremarkable, effectively excluding infectious, inflammatory, and neoplastic etiologies. EEG recordings demonstrated focal-onset seizure activity, correlating with the patient’s recurrent neurological episodes. When interpreted in the context of his remote cranial radiotherapy, this constellation of findings supported the final diagnosis of SMART syndrome, explaining his recurrent stroke-like symptoms, headaches, and seizure activity nearly a decade after treatment for oligodendroglioma.

## Discussion

SMART syndrome is an uncommon delayed complication of cranial irradiation characterized by the acute onset of stroke-like symptoms, migraine-like headache, seizures, and focal neurological deficits in patients with a history of brain radiotherapy. Our patient presented nearly 10 years after receiving 54 Gy focal brain irradiation for an anaplastic oligodendroglioma, highlighting the delayed nature of this syndrome and its relevance to long-term cancer survivorship.

The clinical presentation in SMART syndrome is heterogeneous, often mimicking acute cerebrovascular disease. Headache, hemiparesis, hemianopia, aphasia, and seizures are common manifestations [4,5]. Our patient’s recurrent episodes of left-sided weakness with seizures, in the context of normal CT and MRI findings for acute stroke, underscore the diagnostic dilemma. EEG in this case demonstrated right hemispheric epileptiform activity with secondary generalization, consistent with the recognized epileptogenic potential of SMART syndrome [10]. Importantly, cerebrospinal fluid analysis and autoimmune/paraneoplastic panels were negative, excluding infectious and inflammatory mimics. With the EEG finding the hemiplegic migraine is ruled out. 

Neuroimaging plays a pivotal role in differentiating SMART from other conditions. The hallmark MRI finding is unilateral, transient, gyriform cortical enhancement with corresponding T2/FLAIR hyperintensity sparing the white matter [2,3]. However, in our case, serial MRI scans only demonstrated chronic periventricular ischemic changes, without the typical cortical enhancement. This atypical imaging presentation has been previously described, where clinical suspicion and exclusion of alternative diagnoses remain central to diagnosis [12,14].

The exact mechanism of SMART syndrome remains incompletely understood and is likely multifactorial. Proposed mechanisms include radiation-induced vascular endothelial injury, impaired cerebral autoregulation, demyelination, and delayed mitochondrial dysfunction leading to neuronal hyperexcitability [3,8]. Some parallels have been drawn with posterior reversible encephalopathy syndrome (PRES) and cortical spreading depression observed in migraine and epilepsy [10]. The occurrence of symptoms more than a decade after irradiation in our patient supports the hypothesis of progressive radiation-induced neurotoxicity culminating in episodic neurological dysfunction.

Although SMART syndrome is often described as reversible, recovery may be incomplete, and recurrences are reported in up to one-third of cases. Older age, temporal lobe involvement, and restricted diffusion on MRI are associated with prolonged deficits [14]. Our patient’s recurrent seizures, prolonged confusion, and persistent EEG asymmetry indicate that neurological sequelae may endure despite clinical stabilization. This aligns with reports of SMART-related epilepsy and cognitive decline in long-term survivors [4,5].

There is no standardized treatment protocol for SMART syndrome. Although not used in our case, the corticosteroids are frequently administered and may accelerate recovery, though their efficacy is variable [2]. Antiepileptic drugs (AEDs) are essential in seizure management, as in our case, where lacosamide achieved seizure control after trials of valproate failed. Emerging therapies, including L-arginine infusion, have shown encouraging results in improving both clinical and imaging findings, suggesting a possible role for endothelial or mitochondrial modulation [15]. Supportive therapy, vigilant exclusion of tumor recurrence, and avoidance of unnecessary invasive interventions remain the cornerstones of management [3,10].

This case contributes to the growing literature by highlighting several important aspects: the potential for SMART syndrome to present nearly a decade after cranial irradiation; the diagnostic challenge posed by atypical imaging findings; and the importance of integrating clinical features, EEG, and exclusion of alternative diagnoses in establishing the diagnosis. Furthermore, it underscores the need for heightened awareness among clinicians managing long-term survivors of brain tumors, especially those who received moderate to high-dose radiation therapy.

## Conclusions

SMART syndrome is an uncommon yet clinically important delayed complication of cranial irradiation. Its broad spectrum of neurological manifestations, variable radiologic appearance, and potential for recurrence warrant a high degree of clinical suspicion, particularly in individuals with a remote history of brain radiotherapy who present with new neurological symptoms. Prompt recognition is essential to prevent diagnostic error, minimize unwarranted invasive procedures, and guide appropriate management. This case highlights the importance of sustained multidisciplinary follow-up in neuro-oncology patients long after completion of treatment.
